# Exploring the Willingness of the COVID-19 Vaccine Booster Shots in China Using the Health Belief Model: Web-Based Online Cross-Sectional Study

**DOI:** 10.3390/vaccines10081336

**Published:** 2022-08-17

**Authors:** Dehua Hu, Zhisheng Liu, Liyue Gong, Yi Kong, Hao Liu, Caiping Wei, Xusheng Wu, Qizhen Zhu, Yi Guo

**Affiliations:** 1 Department of Biomedical Information, School of Life Sciences, Central South University, Changsha 410013, China; 2 Xiangya School of Medicine, Central South University, Changsha 410013, China; 3 Shenzhen Health Development Research and Data Management Center, Shenzhen 518028, China; 4 School of Medical Information Engineering, Jining Medical University, Rizhao 276826, China

**Keywords:** COVID-19, booster vaccination, vaccination willingness, health belief model, structural equation model, influencing factor

## Abstract

(1) Objective: To explore Chinese residents’ willingness to receive COVID-19 vaccine booster shots and identify predictors of the level of willingness based on the health belief model (HBM). (2) Methods: The snowball sampling method was used to distribute online questionnaires. A chi-square test was used to analyze the relationship between different variables. The causal relationship between HBM-related factors and booster vaccination intentions was explored by Structural equation modeling (SEM). (3) Results: A total of 898 complete responses were included; 64.3% had already received the booster injection. Most respondents intended to vaccinate themselves, while 16.1% were hesitant. Nearly half of the respondents chose to take the booster injection to support China’s vaccination policy. Using the SEM, perceived susceptibility and perceived barriers were found to have a negative effect on booster vaccination intentions, whereas perceived benefit and cues to action positively affected booster vaccination intentions in the HBM. (4) Conclusions: Factors included in this study have different effects on the willingness to take the COVID-19 booster injections. Sociodemographic characteristics and characteristics of participants’ COVID-19 vaccination have a significant effect on the willingness to receive vaccine booster shots. The HBM constructs can serve as good predictors of the acceptance of vaccine booster shots with the exception of perceived severity, which may benefit health officials in terms of conducting targeted strategies in vaccine programs.

## 1. Introduction

The COVID-19 (coronavirus disease 2019, SARS-CoV-2) continues to be a global pandemic [1]. According to the World Health Organization (WHO), as of 6 June 2022, there were 520 million confirmed cases of COVID-19 and 6.29 million deaths worldwide [2]. Vaccination has been proven to be one of the means of ending the COVID-19 pandemic [3]. Both research and practice have shown that the establishment of herd immunity through large-scale vaccination with COVID-19 vaccines can effectively block the spread of COVID-19 [4]. China officially approved the COVID-19 vaccine to be marketed and made available to the whole population free of charge by the end of 2020. As of 4 June 2022, China reported a total of 3.38 billion doses of COVID-19 vaccines, with a total number of 1.29 billion people vaccinated, and 1.25 billion people had completed the whole course of vaccination, covering 91.56% of the whole population. The number of people who had been vaccinated in the whole process accounts for 89.17% of the whole population [5]. It is evident that Chinese residents are willing to receive the COVID-19 vaccine for the first time, and the actual vaccination rate in China strongly supports this view.

However, several studies [6,7,8] have shown that 6 months after completion of the whole process of vaccination, some recipients showed a decrease in immunity to COVID-19 and an increase in infection rates. Booster vaccines are routinely used for some infectious diseases, either to top up immunity or to update it for new virus variants. After the whole process of immunization followed by booster shots, the neutralization titer in the recipients increased by 25 to 100 times, significantly reducing the infection rate of COVID-19, the incidence of severe cases, and the mortality of the vaccinated [9,10,11,12]. Since the outbreak of COVID-19, the COVID-19 (SARS-CoV-2) variants such as Alpha, Beta, Gamma, Delta, and Omicron have emerged one after another, which has caused COVID-19 to remain a global pandemic, especially the Omicron variant with obvious immune escape, higher reinfection rate, and rapid spread speed. Studies have shown that booster immunization had a neutralizing effect on COVID-19 variants, which can significantly reduce the incidence of severe cases [13,14,15].

As shown in previous studies [16,17], the majority of the vaccinated people in China finished the primary vaccination course with two injections. In addition, according to the COVID-19 booster shots vaccination policy, those who took the one or two injection vaccines during the primary vaccination course are recommended to receive the booster shots to enhance the sustained protective effect of COVID-19 vaccines. As of 4 June, China completed a total of 779.9 million booster immunizations, including 38.34 million sequential booster immunizations [5]. Outbreaks, mainly caused by Omicron variants, have frequently been occurring in many places in China since March 2022, and it is imperative to speed up the coverage of booster immunization as soon as possible.

The health belief model (HBM) is a widely recognized conceptual framework for health behavior [18]. According to this model, in order for an individual to engage in an action to avoid a certain disease, he or she should believe that (1) they are personally susceptible to the condition (the construct of perceived susceptibility), (2) the disease is severe enough to cause concern (perceived severity), (3) taking a particular action is of benefit in reducing susceptibility or severity (perceived benefit), (4) it would not entail overcoming potential barriers (perceived barriers), (5) the confidence in the ability to perform a task or achieve a goal (self-efficacy), and (6) whether there are triggers to acting on the behavior (cues to action) [19]. It is widely used in research related to the interpretation of people’s health behaviors to explore why they engage or do not engage in a wide variety of health-related behaviors. Whether or not to vaccinate is one of the typical health behaviors of people in modern society, and the HBM has been widely used in relevant research, such as influenza vaccination [20,21], HPV (human papillomavirus) vaccination [22,23], and parents’ willingness to vaccinate their children [24,25,26,27]. Since the outbreak of the COVID-19 pandemic, the HBM has been used to predict the public’s vaccination intentions against COVID-19 [28,29,30,31,32,33,34]. Additionally, another study explored the public’s attitude towards vaccination on the basis of the HBM [35]. A systematic review has also proved that the HBM was applicable to this research [36].

There are only a few studies based on the HBM that focus on booster immunization strategies and influencing factors for all suitable groups (people who are over 18 years old and have completed the whole process of vaccination for 6 months [37]) in China [17,38], and little research on the willingness of older adults [39] and factory workers [40] to vaccinate themselves and child caregivers to vaccinate children [41]. This study aims to investigate the willingness and influencing factors of the booster immunization of COVID-19 in China on the HBM by using the structural equation model (SEM) to provide a scientific basis for more targeted publicity and education on booster immunization and expedite the national immunization barrier.

## 2. Materials and Methods

### 2.1. Study Design and Participants

The aim of this study is to explore the Chinese’s willingness to take booster injections on the basis of the HBM. All participants gave their informed consent for inclusion before they participated in the study. The study was conducted in accordance with the Declaration of Helsinki, and the protocol was approved by the Institutional Review Board of the College of Life Sciences, Central South University (Reference No.: 2022-1-23).

We conducted a cross-sectional study based on an online questionnaire with the Wen Juan Wang (https://www.wenjuan.com/ accessed on 4 May 2022), which is a network platform for the compilation of questionnaires. Participants were recruited online at random and asked to distribute questionnaires to other participants with the snowball sampling method. The online questionnaire was shared through social software such as WeChat and QQ in the form of both web links and QR codes. In order to encourage the respondents to complete the questionnaire, a red envelope with RMB 600 (about USD 90) was randomly sent. Exclusion criteria for enrollment were (1) living in mainland China for less than 6 months, (2) inability to understand the content of the questionnaire, and (3) refusing to participate in the study.

The questionnaire began with a brief description of the purpose of the study, the number of questions, the estimated time participants may spend on the questionnaire, the privacy and security assurance statement, and informed consent to participate in the study. There were 27 questions in the questionnaire, consisting of the following sections: (1) sociodemographic characteristics, including age, gender, educational level, living area, permanent residence, income situation, medical insurance, occupation, current location, and the risk level of the area (divided into three categories from low to high), (2) the information on COVID-19 vaccines, such as the type of vaccine, manufacturer of vaccines, completion time and perceived effects from the primary series of COVID-19 vaccination, whether to meet the requirements for a booster vaccination and get booster shots, the intention to get booster injection of their friends, families, and themselves, (3) measurement of HBM variables, including perceived severity, perceived susceptibility, perceived benefits, perceived barriers, self-efficacy, and cues to action. Answers of intentions to receive injections and measurement of HBM variables were on 5-point Likert scales ranging from “strongly disagree” (scored 1 point) to “strongly agree” (scored 5 points).

The questionnaire was collected from 21 January 2022 to 28 January 2022. Individual IP addresses were limited to submitting answers only once in order to prevent repeated submissions. The sample size of 987 individuals was determined using the sample size statistical formula for cross-sectional surveys: initial sample size *n* = [(z^2^ * p * q)]/d^2^. Based on p = proportion of the population who are interested in being vaccinated boosters = 70% (according to the trial test results), z = 1.96 equivalent to 95% confidence interval, d = error not more than 3%, and a 10% non-response rate. Before distributing the questionnaire to the general public, a trial test was conducted with groups representing 5% of the total sample size. Based on the results of the exploratory factor analysis and participant feedback, the questionnaire was revised to ensure its validity and comprehensibility. In the formal survey, we received a total of 1014 questionnaires, of which 898 were valid after excluding those that met exclusion criteria. Among the 116 excluded questionnaires, 8 were caused mainly due to age. The sample recovery rate was 88.56%. Our inclusion criteria for analysis were: (1) the first round of vaccination has been completed, (2) informed consent and voluntary participation in this study. Exclusion criteria for analysis were: (1) answering the questionnaire takes less than 90 s, (2) questionnaires with missing values and outliers, (3) incorrect answer to the general knowledge question, (4) consistent answers to questions designed by 5-point Likert scales.

### 2.2. Statistical Analysis

A chi-square test was used to analyze the relationship between sociodemographic variables, vaccination status-related variables, and booster vaccination intentions, respectively. The correlation between HBM-related factors and booster vaccination intentions was explored by Structural Equation Model. Cronbach’s α test and composite reliability (CR) were used to test the reliability of HBM measures, while confirmatory factor analysis (CFA) and the average variance extracted (AVE) to validity. This research adopted the SPSS 25.0 (IBM Corp, New York, NY, USA, 2017) for statistical analysis and the chi-square test. AMOS 23.0 (IBM Corp, New York, NY, USA, 2015) was used to evaluate the structural model. The statistical significance was defined as *p* < 0.05.

## 3. Results

### 3.1. Sociodemographic Characteristics

This study included 898 participants, 381 (42.4%) were male and 517 (57.6%) were female. The age range of the participants was 12 to 63, with the largest percentage (75.3%) in the 19–30 group. Most of them were urban residents (n = 715, 79.6%) and were educated with an associate college degree and above (79.9%). Their income was mainly below RMB 8000 (about USD 1185.6). Students and other corporate employees made up the largest proportions, 26.9% and 24.6%, respectively; Medical workers (6.3%) and farmers (2.6%) had the smallest proportions. Most respondents were in areas with a low risk level for the relatively safe environment of the COVID-19 pandemic in China. The overwhelming majority of participants (90.5%) had health insurance; there were significant differences in booster vaccination willingness among participants of different genders, educational levels, occupations, and locations. The exact numbers and characteristics of the respondents are presented in Table 1.

### 3.2. Characteristics of Participants’ COVID-19 Vaccination

Among the participants, 577 respondents (64.3%) had received booster shots before this research. A total of 87.6% of participants met the requirements for booster shots, and the requirements for vaccination booster shots were (1) at least 18 years old and (2) 6 months or more after completing the full course of vaccination (including 1 dose, 2 doses). Characteristics of participants’ COVID-19 vaccination are reported in detail in Table 2. Participants who chose the 2-dose option had the highest proportion (54.6%). Their preference for the manufacturer of vaccines was Sinovac Biotech Co., Ltd. based in Beijing, China, with a percentage of 57.2%. A total of 81.1% of the respondents considered the primary series of COVID-19 vaccination to be effective or moderate, while only 19 (2.1%) deemed it to be less effective in terms of protection. The willingness of friends and family members to receive booster shots was very high (83.3% and 83.9%, respectively), and the proportion of moderate willingness was 12.6% and 12.2%, respectively. The statistical analysis indicated that the type of vaccine, manufacturer of the vaccine, perceived effects from the primary series, and willingness of friends and family members to receive booster shots were significantly related to booster vaccination intentions.

A total of 48.9% of those who had received the booster shot were vaccinated to support vaccination efforts in China, followed by those who got booster shots (26.9%) to further enhance the protective effect of the COVID-19 vaccines. Other reasons for receiving booster shots are shown in Table 3.

### 3.3. HBM Predictive Factors of COVID-19 Booster Vaccination

Table 4 shows the reliability and validity of the HBM measurement; the value of AVE ranges between 0.553 and 0.742, which is higher than 0.50. Values of CR were between 0.710 and 0.919, which were higher than 0.70, indicating that constructs had good convergent validity. The correlations of the constructs were smaller than the square root of the average variance extracted from each construct, indicating discriminant validity, as shown in Table 5.

Confirmatory factor analysis was used to evaluate the fit of the model. The model-fit indices were as follows: χ^2^/df = 2.474, CFI (Comparative Fit Index) = 0.976, GFI (Goodness-of-Fit Index) = 0.957, TLI (Tucker–Lewis Index) = 0.971, and RMSEA (Root-Mean-Square Error of Approximation) = 0.041, which indicates that the research model fitted the collected data well (CFI > 0.9, GFI > 0.9, TLI > 0.9, RMSEA < 0.05).

**Figure 1:**
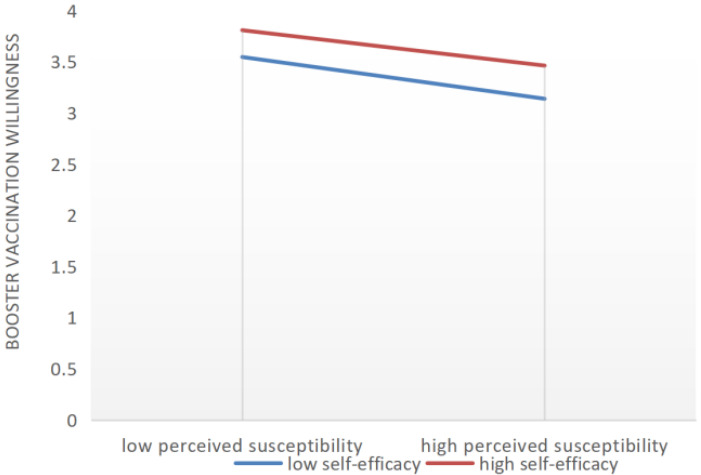
The moderating effects of self-efficacy on the relationship between perceived susceptibility and booster vaccination intention.

**Figure 2:**
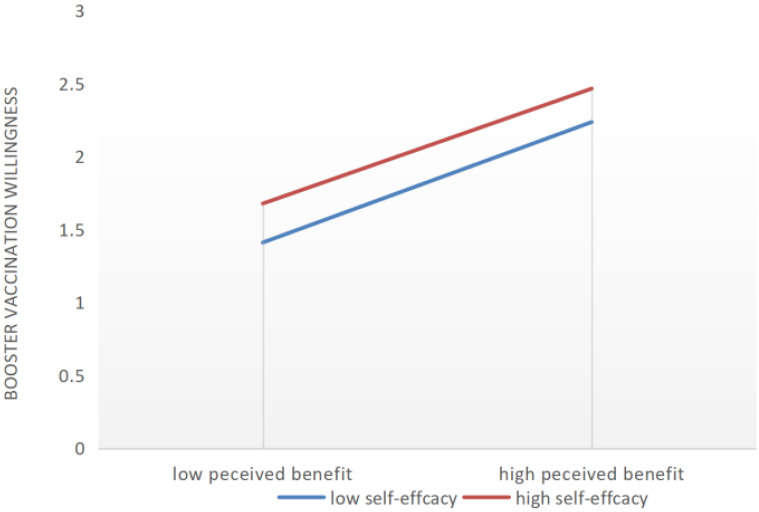
The moderating effects of self-efficacy on the relationship between perceived benefits and booster vaccination intentions.

**Figure 3:**
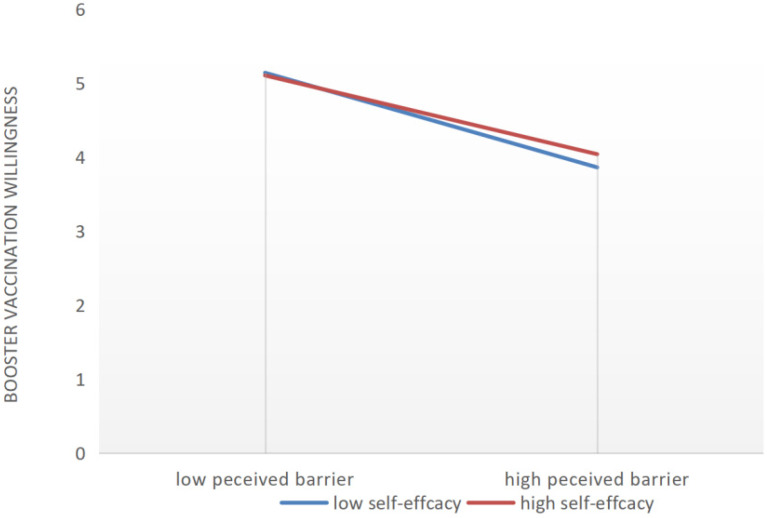
The moderating effects of self-efficacy on the relationship between perceived barriers and booster vaccination intentions.

The path coefficients of the SEM of booster vaccination intentions are shown in Table 6. Among HBM predictive factors, perceived susceptibility and perceived barriers had a negative effect on booster vaccination intentions, whereas perceived benefits and cues to action positively affect booster vaccination intentions. In addition, the relationship between perceived susceptibility, perceived barriers, and perceived benefits on booster vaccination intentions were moderated by self-efficacy, and the corresponding *p*-value was less than 0.001 after examination. With high self-efficacy, the negative impact of perceived susceptibility on booster vaccinations was reduced, and the positive effect of perceived benefits on booster vaccination was weakened. Perceived barriers’ negative impact on booster vaccination was attenuated by the presence of high self-efficacy (Figure 1, Figure 2 and Figure 3). In Figure 1, Figure 2 and Figure 3, the y-axis indicates the change trend of the willingness to receive a booster. Taking zero as the origin, the higher the y-axis, the stronger the willingness to receive a booster.

## 4. Discussion

COVID -19 variants are rampant all over the world, and the promotion and popularization of vaccine booster injections, either homologous or heterologous, is an effective means to prevent SARS-CoV-2 transmission [42,43,44]. The nonnegligible challenge will be to enhance the communication between healthcare providers and the public to overcome vaccine hesitancy and make vaccination services accessible to all people, older and vulnerable people in particular [45]. However, there are few related surveys on the willingness to receive booster shots [46].

In general, residents in China have a strong sense of epidemic prevention after an arduous fight against COVID-19. The share of respondents who said they were willing to receive booster shots is relatively high, up to 83.9%, including 64.3% who had already been vaccinated with the booster shots at the time of data collection. This finding is much higher than the level among urban employees from a megacity in eastern China with a rate of 60.1% [46], and closely resembles the levels of primary vaccine acceptance surveyed in China [47], Saudi Arabia [48], and Italy [49], reaching more than 80%. It is worth noting that 10.2% of the respondents were hesitant about whether to receive the booster shot, and 5.9% were unwilling to accept it in our study, which needs our close attention. There are at least three issues contributing to vaccine hesitancy. First, the speed at which vaccines have been developed has raised concerns that the trials were rushed and regulatory standards relaxed [50]. Second, the novelty of vaccine research and development has also sparked hesitancy [51]. Third, conspiracy theories about COVID-19 vaccines are being widely circulated on unregulated social media platforms [52,53], sometimes by highly organized anti-vaccination groups [54,55,56].

This study showed a significantly positive correlation between sociodemographic characteristics (including gender, educational level, occupation, and the risk level of the area) and the willingness to receive booster shots. In addition, relating to the free vaccination policy and its thorough implementation, there was no correlation between monthly income, whether to purchase medical insurance, and willingness to vaccinate booster shots, which was consistent with that noted in a study about acceptance of the primary series in China [16,57]. Additionally, the analysis of simple correlation indicated a significant positive correlation between the type of vaccines, manufacturer of vaccines, and the willingness to receive booster shots, reflecting the public’s trust in the safety of China’s COVID-19 vaccines. To a certain extent, the result shows there is a linear relationship between the level of perceived effects from the primary series, the willingness of friends and family members to receive booster shots, and the intensity of willingness to receive booster shots, which suggests the effectiveness of China’s COVID-19 primary vaccines. While quite a few people may take the vaccination out of conformity psychology, it is still essential to educate the public with vaccine knowledge to further improve their professionalism.

Regarding the reasons for receiving booster shots, 48.9% of respondents had an inoculation to support the vaccination policy in China and 26.9% to further enhance the protective effect of COVID-19 vaccines, indicating that Chinese residents generally tend to agree with the government’s policy of promoting vaccine booster shots both to affirm the hard work of the government and strengthen their self-protection. This also proved that the extensive publicity about booster shots is effective in China.

The study used the HBM, which is useful for studying the uptake of medical services and interventions [48]. The results revealed that all the constructs adopted in the HBM were shown to be good predictors of willingness to receive booster injections with the exception of perceived severity, which did not significantly predict behaviors, showing no difference with other studies [48,57]. Self-efficacy reflects an individual’s perception of his or her ability to perform a behavior successfully, which can modify the relationship between perceived susceptibility, perceived benefits, perceived barriers, and willingness to receive the booster shot. Cues to action were what triggered the individual to engage in a behavior. The significantly positive correlations between perceived benefits, cues to action, and the willingness to receive the booster shots were proved. In this study, respondents showed the opposite tendency. The more perceived susceptibility the groups had, the more reluctant they were to receive the booster shot, indicating that there was a negative correlation between perceived susceptibility and willingness to receive the booster shot. We hypothesized that self-susceptible respondents worry about infecting themselves in the inoculation environment where there are dense queues waiting to be vaccinated, which indirectly led to the groups of perceived susceptibility hesitation. However, this hypothesis still needs further testing. Even so, it’s a noteworthy reminder that the government and other public organizations should strengthen the publicity of knowledge about vaccine booster shots and self-protective measures to dispel doubts, which may help to enhance their willingness to get inoculated.

## 5. Conclusions

In this cross-sectional study, an online questionnaire based on the HBM was sent to participants selected by snowball sampling. Based on a sample size of 878 respondents, this study tested the relationship between various variables and the willingness to receive booster shots through the internet questionnaire survey. In this study, we found that factors included in this study have different effects on the willingness to take the COVID-19 booster injections, including sociodemographic characteristics (including gender, educational level, occupation, and the risk level of the area), characteristics of participants’ COVID-19 vaccination (including the type of vaccine, manufacturer of the vaccine, perceived effects from the primary series, willingness of friends and family members to receive booster shots). The HBM constructs can serve as good predictors of the acceptance of vaccine booster shots, with the exception of perceived severity.

## 6. Limitations and Prospects

There are some limitations to this study. (1) As this survey was implemented online, our respondents were mainly composed of young people, who are more likely to access our questionnaires on the internet. Additionally, participants were selected using the snowball sampling method. This may affect the representativeness of the respondents to a certain extent. (2) This study was mainly based on the Chinese context, which means vaccinations (including booster shots) are free regardless of whether medical insurance is purchased or not. The willingness to receive the vaccination is affected by various factors, including policy and legislation, and the influencing factors need further study. In China, people have strong political sensitivity and keep relatively synchronized with government policies. (3) Our results were not in line with people’s common sense that there is a negative correlation between perceived susceptibility and willingness to receive the booster shot, and the underlying causes need further excavation and analysis.

Future studies need to break through this limitation and make more representative national-level studies of cross-country studies that include all age groups. Despite these limitations, our research still depicts the acceptance of COVID-19 vaccine booster shots in China. In fact, this study clarifies the misunderstandings of Western countries about China’s administrative compulsory vaccination. The sample data shows that the main reasons for people to receive boosters are to support national policies and protect their own health, rather than mandatory booster shots.

## Figures and Tables

**Table 1:** Sociodemographic characteristics of the sample (n = 898).

Variables	Total	Willingness to Receive Vaccine Booster Shots			Chi-Square	*p*-Value
	n (%)	Intended (83.9%)	Undecided (10.2%)	Unwilling (5.9%)		
Gender						
Female	381 (57.6)	323 (84.8)	48 (12.6)	10 (2.6)	15.69	<0.001
Male	517 (42.4)	430 (83.2)	44 (8.5)	43 (8.3)		
Age group						
18 and below	94 (10.5)	77 (81.9)	13 (13.8)	4 (4.3)	10.39	0.109
19–30	676 (75.3)	559 (82.7)	73 (10.8)	44 (6.5)		
31–40	93 (10.4)	87 (93.5)	2 (2.2)	4 (4.3)		
Above 40	35 (3.9)	30 (85.7)	4 (11.4)	1 (2.9)		
Living area						
Urban	715 (79.6)	604 (84.5)	68 (9.5)	43 (6.0)	2.08	0.353
Rural	183 (20.4)	149 (81.4)	24 (13.1)	10 (5.5)		
Educational background						
Junior high school and below	49 (5.5)	42 (85.7)	4 (8.2)	3 (6.1)	34.22	<0.001
High school	131 (14.6)	104 (79.4)	17 (13.0)	10 (7.6)		
Associate college	230 (25.6)	206 (89.6)	11 (4.8)	13 (5.7)		
Bachelor’s degree	387 (43.1)	333 (86.0)	36 (9.3)	18 (4.7)		
Master’s degree and above	101 (11.2)	68 (67.3)	24 (23.8)	9 (8.9)		
Monthly income (yuan)						
Under 5000	354 (39.4)	295 (83.3)	43 (12.1)	16 (4.5)	6.72	0.347
5000–8000	306 (34.1)	262 (85.6)	23 (7.5)	21 (6.9)		
8000–12,000	175 (19.5)	146 (83.4)	17 (9.7)	12 (6.9)		
Over 12,000	63 (7.0)	50 (79.4)	9 (14.3)	4 (6.3)		
Occupation						
Medical personnel	57 (6.3)	53 (93.0)	2 (3.5)	2 (3.5)	38.3	<0.001
Civil Service	132 (14.7)	111 (84.1)	10 (7.6)	11 (8.3)		
Service industry personnel	162 (18.0)	139 (85.8)	6 (3.7)	17 (10.5)		
Other corporate employees	242 (26.9)	200 (82.6)	32 (13.2)	10 (4.1)		
Teachers	39 (4.3)	34 (87.2)	1 (2.6)	4 (10.3)		
Students	221 (24.6)	179 (81.0)	36 (16.3)	6 (2.7)		
Farmers	23 (2.6)	20 (87.0)	2 (8.7)	1 (4.3)		
Others	22 (2.4)	17 (77.3)	3 (13.6)	2 (9.1)		
Risk level of the area						
Low Risk	778 (86.6)	657 (84.4)	85 (10.9)	36 (4.6)	20.41	<0.001
Medium Risk	101 (11.2)	79 (78.2)	7 (6.9)	15 (14.9)		
High Risk	19 (2.1)	17 (89.5)	0 (0.0)	2 (10.5)		
Medical insurance						
Yes	813 (90.5)	687 (84.5)	78 (9.6)	48 (5.9)	3.98	0.136
No	85 (9.5)	66 (77.6)	14 (16.5)	5 (5.9)		

**Table 2:** Characteristics of participants’ COVID-19 vaccination. (n = 898).

Variables	Total	Willingness to Get COVID-19 Vaccine Boosters			Chi-Square	*p*-Value
	n (%)	Intended (83.9%)	Undecided (10.2%)	Unwilling (5.9%)		
Type of vaccines (Classified by the times of injections)						
1 injection	23 (2.6)	15 (65.2)	3 (13.0)	5 (21.7)	18.18	0.001
2 injections	490 (54.6)	399 (81.4)	59 (12.0)	32 (6.5)		
3 injections	385 (42.9)	339 (88.1)	30 (7.8)	16 (4.2)		
Manufacturer of vaccines						
Wuhan Institute of Biological Products in Wuhan, China	88 (9.8)	77 (87.5)	8 (9.1)	3 (3.4)	27.11	0.007
Beijing Institute of Biological Products Co., Ltd. in Beijing, China	121 (13.5)	102 (84.3)	10 (8.3)	9 (7.4)		
Sinovac Biotech Co., Ltd. in Beijing, China	514 (57.2)	445 (86.6)	43 (8.4)	26 (5.1)		
Tianjin Cansino Biotechnology Inc. in Tianjin, China	41 (4.6)	30 (73.2)	5 (12.2)	6 (14.6)		
Anhui Zhifei Longcom Biopharmaceutical Co., Ltd. in Anhui, China	40 (4.5)	27 (67.5)	10 (25.0)	3 (7.5)		
Other manufacturers	16 (1.8)	11 (68.8)	4 (25.0)	1 (6.3)		
No knowledge of the manufacturer	78 (8.7)	61 (78.2)	12 (15.4)	5 (6.4)		
Perceived effects from the primary series						
Very high	392 (43.7)	373 (95.2)	9 (2.3)	10 (2.6)	259.3	<0.001
High	372 (41.4)	323 (86.8)	37 (9.9)	12 (3.2)		
Moderate	115 (12.8)	50 (43.5)	44 (38.3)	21 (18.3)		
Low	13 (1.4)	4 (30.8)	2 (15.4)	7 (53.8)		
Very low	6 (0.7)	3 (50.0)	0 (0.0)	3 (50.0)		
Friends’ willingness to receive booster shots					697.0	
Very high	452 (50.3)	438 (96.9)	7 (1.5)	7 (1.5)		
High	296 (33.0)	271 (91.6)	18 (6.1)	7 (2.4)		<0.001
Moderate	113 (12.6)	35 (31.0)	66 (58.4)	12 (10.6)		
Low	19 (2.1)	8 (42.1)	1 (5.3)	10 (52.6)		
Very low	18 (2.0)	1 (5.6)	0 (0.0)	17 (94.4)		
Family members’ willingness to receive booster shots						
Very high	505 (56.2)	492 (97.4)	4 (0.8)	9 (1.8)	528.9	<0.001
High	249 (27.7)	215 (86.3)	27 (10.8)	7 (2.8)		
Moderate	110 (12.2)	39 (35.5)	55 (50.0)	16 (14.5)		
Low	20 (2.2)	7 (35.0)	5 (25.0)	8 (40.0)		
Very low	14 (1.6)	0 (0.0)	1 (7.1)	13 (92.9)		

**Table 3:** Reasons for receiving booster shots (n = 577).

Reasons for Receiving Booster Shots	n (%)
Supporting vaccination policy in China	282 (48.9)
Vaccination required by workplace or school	73 (12.7)
Further enhancing the protective effect of the COVID-19 vaccine	155 (26.9)
Fears of contracting a mutant strain of the coronavirus despite vaccination	57 (9.9)
Chose to receive the booster vaccination because of others’ vaccination	10 (1.7)

**Table 4:** Reliability and validity of HBM measures.

Items	Cronbach’s α	AVE ^1^	CR ^2^
Perceived Severity	0.822	0.565	0.834
Perceived Susceptibility	0.705	0.553	0.710
Perceived Benefits	0.876	0.641	0.877
Perceived Barriers	0.917	0.742	0.919
Self-Efficacy	0.832	0.621	0.831
Cues to Action	0.836	0.631	0.837

^1^ AVE is the average variance extracted from the model; ^2^ CR is composite reliability.

**Table 5:** Correlation matrix of HBM measures. (n = 898).

	Perceived Severity	Perceived Susceptibility	Perceived Benefits	Perceived Barriers	Self-Efficacy	Cues to Action
Perceived Severity	0.752 ^1^					
Perceived Susceptibility	0.385	0.744				
Perceived Benefits	0.284	0.352	0.801			
Perceived Barriers	0.225	0.104	−0.213	0.861		
Self-Efficacy	0.305	0.315	0.655	−0.101	0.788	
Cues to Action	0.302	0.299	0.670	−0.107	0.723	0.794

^1^ The value of the diagonal is the square root of the average variance extracted from each construct.

**Table 6:** Effect of HBM variables on public COVID-19 booster vaccination intentions.

Paths	C.R. ^1^	Unstandardized Path Coefficients ^2^	Standardized Path Coefficients ^2^	*p*-Value
Perceived Severity → Booster Vaccination willingness	−0.561	−0.031	−0.023	0.575
Perceived Susceptibility → Booster Vaccination willingness	−2.207	−0.125	−0.109	0.027
Perceived Benefit → Booster Vaccination willingness	2.102	0.233	0.148	0.036
Perceived Barriers → Booster Vaccination willingness	−4.053	−0.139	−0.151	<0.001
Cues to Action → Booster Vaccination willingness	2.977	0.438	0.308	0.003

^1^ C.R.: Critical ratio, dividing the regression weight estimate by the estimate of its standard error gives. ^2^ Path coefficient: the value that the dependent variable goes up by when the independent variable goes up by 1.

## Data Availability

The data that support the findings of this study are available from the corresponding author upon reasonable request.
